# Variations in Reactive Oxygen Species Generation by Urban Airborne Particulate Matter in Lung Epithelial Cells—Impact of Inorganic Fraction

**DOI:** 10.3389/fchem.2020.581752

**Published:** 2020-12-17

**Authors:** Olga Mazuryk, Grazyna Stochel, Małgorzata Brindell

**Affiliations:** Faculty of Chemistry, Jagiellonian University, Kraków, Poland

**Keywords:** air pollution, particulate matter PM, reactive oxygen species, inorganic fraction of PM, treatment protocol

## Abstract

Air pollution is associated with numerous negative effects on human health. The toxicity of organic components of air pollution is well-recognized, while the impact of their inorganic counterparts in the overall toxicity is still a matter of various discussions. The influence of airborne particulate matter (PM) and their inorganic components on biological function of human alveolar-like epithelial cells (A549) was investigated *in vitro*. A novel treatment protocol based on covering culture plates with PM allowed increasing the studied pollutant concentrations and prolonging their incubation time without cell exposure on physical suffocation and mechanical disturbance. PM decreased the viability of A549 cells and disrupted their mitochondrial membrane potential and calcium homeostasis. For the first time, the difference in the reactive oxygen species (ROS) profiles generated by organic and inorganic counterparts of PM was shown. Singlet oxygen generation was observed only after treatment of cells with inorganic fraction of PM, while hydrogen peroxide, hydroxyl radical, and superoxide anion radical were induced after exposure of A549 cells to both PM and their inorganic fraction.

## Introduction

Poor air quality is one of the global health problems, since it affects around 91% of the world's population (World Health Organization) (http://www.who.int). Numerous reports confirm that exposure to particulate matter (PM) increases the risks of respiratory tract, cardiovascular, immunological, and other diseases (Araujo, 2011; Selmi et al., 2012; Kelly and Fussell, 2017; Zhang et al., 2018; Shen and Lung, 2020; Yang et al., 2020; Wang et al., 2020a). Despite the fact that the health effects of air pollution have been recognized since the last century, the progress in research is still not satisfied and it might arise from the difficulty to reliably assess the impact of air pollutants on human health. The effects of chronic exposure are usually observed only after several or even dozens of years, while acute contact often does not result in detectable biological alterations (Bräuner et al., 2007; Han et al., 2020). The bio-accumulative nature of pollutions makes it possible to reach up the threshold level that is harmful to human health (Ali et al., 2019) and safe levels of exposure are difficult to establish (Liu et al., 2018). Therefore, small constantly applied disturbances to cellular homeostasis can lead to pathological changes and result in the development of various diseases.

Air pollution consists of multi-component and multi-phase chemical systems that include traces of transition and main group metals and compounds (minerals, carbonaceous species, inorganic ions), various gaseous components (O_2_, O_3_, NO_x_, HS^−^/H_2_S, CO, CO_2_, SO_2_), and organic contaminations [polycyclic aromatic hydrocarbons (PAHs), chlorinated pesticides, quinones, and biological materials such as viruses, endotoxins, cell fragments]. All components are constantly interacting in gas–liquid, liquid–solid, and gas–liquid–solid interfaces. The health problems originating from air pollution are mainly linked to PM of <0.1 and 2.5 μm in diameter (denoted as PM_0.1_ and PM_2.5_, respectively), which can easily penetrate the respiratory tract and accumulate more efficiently (Gangwar et al., 2020; Leikauf et al., 2020; Manigrasso et al., 2020; Novák et al., 2020). PM is a complex mixture of particles with a wide range of size and diverse composition, depending on the place and time of sample collection (Shao et al., 2018). The natural sources contributing to high PM emission are windblown dust, volcano eruptions, wildfires, and sea salt aerosols, while industrial, automobile, and construction/demolition sectors are the major anthropogenic contributors to air pollution (Ali et al., 2019). Numerous worldwide government health regulations aimed at the improvement of air quality have shifted PM emission contribution from different sources, for example, from being dominated by coal burning to a mix of vehicle and stationary combustion emissions (Shao et al., 2018). Each PM source generates several potential toxic elements. Such large heterogeneity of the naturally collected PM samples can impede the evaluation of the biological effects of PM and obstruct establishing of underlying toxicological mechanisms. Nowadays, standard reference materials for urban air pollutant are commercially available and widely used as the reference samples in various biological and chemical studies (Park et al., 2013; Courtois et al., 2014; Jiang et al., 2014; Lee et al., 2016; O'Driscoll et al., 2019; Dijkhoff et al., 2020).

In our work, we are focusing on the evaluation of the impact of the inorganic fraction of PM (in particular redox-active transition metal elements) on cells by using the standard reference material for urban air pollutants deprived of organic components. The toxicity of organic compounds is usually based on specific interaction with biomolecules and is widely described in the literature (Hanzalova et al., 2010; Oh et al., 2011; Falco et al., 2017; Qi et al., 2020; Wang et al., 2020b). Among the most hazardous chemical compounds are PAHs, which have carcinogenic, mutagenic, and/or teratogenic effects. They are lipophilic and can easily penetrate tissues, interacting with different enzymes (such as cytochrome P450), producing reactive toxic metabolites and genotoxic DNA adducts, and activating different biological pathways (Baulig et al., 2003; Chew et al., 2020; Longhin et al., 2020). The activity of inorganic PM is predominantly based on its redox chemistry and ability to induce oxidative stress (Kasprzak, 2002; Kawanishi et al., 2002; Visalli et al., 2015; Cui et al., 2019; Haghani et al., 2020; Pardo et al., 2020; Samet et al., 2020). Several studies were performed to establish an association between toxic effects of air pollution and transition metal components of PM such as Fe, V, Cr, Cu, and Zn (Ali et al., 2019; Valacchi et al., 2020). In our opinion, greater attention should be paid to the inorganic components, since they can catalyze different oxidation reactions, whereas organic pollutants can produce reactive oxygen species (ROS) mainly in a stoichiometric way (Risom et al., 2005; Andersson et al., 2009). Different mechanisms of pathological cellular response induced by organic and inorganic fractions of PM are postulated, and serious debate on their roles has been going on in the scientific community in recent years (Stone et al., 2003; Lu et al., 2014; Huang et al., 2015; Kim et al., 2018; Badran et al., 2020). Several reports disclosed the predominant role of organic pollutants due to their ability to induce a pro-inflammatory response in murine macrophages or bronchial epithelial cells (Baulig et al., 2003, 2009; Billet et al., 2007; Gawda et al., 2018; Wang et al., 2018). Alternatively, there are data that indicate the limited importance of endotoxin and organic fraction of air pollutants on inflammatory response to PM, suggesting the involvement of other components of PM, which need to be identified (Herseth et al., 2017). Furthermore, synergistic and antagonistic interactions between PM components could significantly alter their redox properties (Gao et al., 2020). Significant discrepancy in the current literature state of knowledge might be related to the different experimental protocols applied to the performed research. The studied air pollutant samples varied by not only chemical/physicochemical characteristics (different organic and inorganic contents, particle size, zeta potential, and others) but also different experimental models, exposure time, and concentrations used. Hence, direct comparison of the results received for the inorganic and organic PM fractions is challenging.

**Scheme 1:**
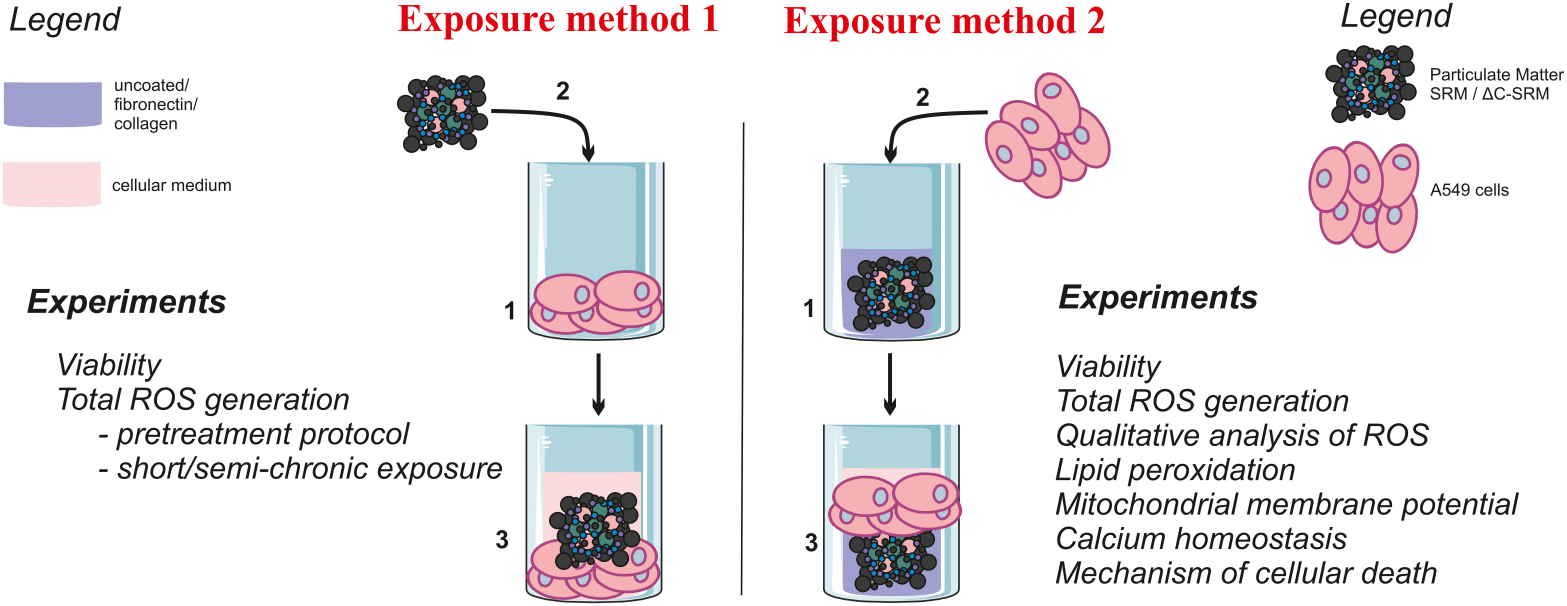
The graphical representation of the used exposure methods and performed experiments.

To improve our knowledge on the direct mechanisms involved in PM-induced toxicity with a particular interest on the role of inorganic components, the influence of PM on type II human alveolar-like epithelial cell line A549 was analyzed *in vitro*. Several biological parameters such as cells' viability and mechanism of cellular death as well as ROS production, mitochondrial potential, and cytosolic calcium homeostasis were evaluated after exposure to PM samples, both intact (inorganic and organic PM fractions) and plasma treated (mostly inorganic fraction). Additionally to standard exposure protocol involving cell treatment with a suspension of PM, the new protocol based on covering culture plates with PM and coating them with adhesion proteins was applied (Scheme 1). Limited contact of the biggest particles (microparticles) of PM with cells allowed the avoidance of mechanical disturbance of cells. This approach enables a focus on the evaluation of the impact of PM as well as its dissolved compounds on cells without additional contribution from dying physically disturbed cells. Particular attention was paid to use small non-toxic concentrations of PM to avoid false-positive results caused by a fraction of dead cells as well as fluorescent probe oxidation caused by PM.

## Materials and Methods

### Sample Preparation

Urban PM sample SRM 1648a (encoded as SRM) was purchased from the National Institute of Standards and Technology (USA). This sample is composed of PM collected in the St. Louis, MO, area over a 1-year period (1976–1977). Among organic constituents, SRM contained PAHs, nitro-substituted PAHs, polychlorinated biphenyls (PCBs), and chlorinated pesticides. Analysis of the inorganic fraction revealed, among others, components containing Fe, Mn, Al, Ca, Cr, Ti, Pb, Mg, and others. The sample was fully characterized by numerous analytical techniques, and its specification is available in the manufacturer's certificate. The particle size varies from 0.2 to over 100 μm, with the predominance of particles with a hydrodynamic diameter of 5–20 μm.

The Plasma Zepto system (Diener Electronic GmbH) was used for the removal of organic compounds present in the SRM sample. Low-temperature plasma treatment is a well-known technique for the elimination of organic contamination, which are oxidized, converted into volatile products, and sucked by pump. Samples (17–20 mg) were treated with a low-temperature plasma for 120 min at 100 W power. Content of carbon was determined by the elementary analysis (Ewlementar, Vario Micro Cube) and total organic carbon (TOC) analyzer (Schimadzu, TOC-V series). Upon plasma treatment, the carbon content decreased from 14% in the SRM sample to 1.8% in the plasma-treated sample (encoded as ΔC-SRM) (Mikrut et al., 2018). Plasma treatment did not induce significant changes in the sample morphology or aggregation (Mikrut et al., 2018).

PM samples were weighed on a high-precision microbalance, and stock suspensions were sonicated for a few minutes before use. Fresh suspensions were prepared before each experiment.

### Cell Culture Conditions

Biological studies were performed using type II human alveolar-like epithelial cell line A549. Cells were maintained in Dulbecco's modified Eagle's medium (DMEM) supplemented with 10% fetal bovine serum (FBS) and 1% antibiotics (penicillin 100 units/ml, streptomycin 100 μg/ml) and were routinely cultured at 37°C in a humidified incubator in a 5% CO_2_ atmosphere. Cells were seeded on unmodified plates 24 h before performing the experiments, typically with a density of 3 × 10^4^ cells per cm^2^. If not stated otherwise, cells were exposed to PM for 24 h (added as a suspension in medium), followed by washing with phosphate buffered saline (PBS) (exposure method 1, Scheme 1). After that, an appropriated test was applied. Alternatively, cells were seeded on plates that were pre-prepared by covering them with PM samples (as described in the following paragraph) and kept for 24 h (exposure method 2, Scheme 1). All experiments were performed at least in triplicate and repeated three times. The mean values ± standard error of the mean (SEM) were calculated.

### Covering Plates With Particulate Matter Samples (Exposure Method 2)

To cover the plates with PM samples, SRM, or ΔC-SRM was suspended in cold methanol and added to the wells to achieve different concentrations up to 625 μg/cm^2^. After methanol evaporation, some plates were additionally coated with fibronectin from human plasma or collagen I from calf skin (Sigma). Fibronectin and collagen were diluted to 25 μg/ml with sterile water, and 50-μl solutions were added to the wells of 96-well-plates. Plates were kept at 4°C overnight, and then they were directly used for seeding cells.

### Cell Viability

The evaluation of the A549 cell viability after exposure to PM samples was conducted using 3-[4,5-dimethylthiazol-2-yl]-2,5-diphenyltetrazolium bromide (MTT) or resazurin assays. The MTT assay is based on the reduction of yellow tetrazolium salt (MTT) by dehydrogenases of metabolically active cells into purple water-insoluble formazan dye. The concentration of dye determined spectrophotometrically after dissolution in an organic solvent is proportional to the number of live cells. Cell viability was quantified measuring absorbance at 565 nm using 700 nm as a reference wavelength (Tecan Infinite 200 microplate reader). Resazurin test is based on the reduction of blue and non-fluorescent substrate (resazurin) to a pink and highly fluorescent product (resorufin) by the live cells. Cell viability was quantified at 605 nm using 560-nm excitation light (Tecan Infinite 200 microplate reader). Experiments were performed in triplicate and repeated at least three times to get the mean values ± SEM. The viability was calculated with respect to the control seeded on uncoated/coated plate without PM samples. The viability was determined after incubation of cells with PM samples prepared as suspension in a medium [without or with 2/10% fetal bovine serum (FBS)] at concentrations up to 500 μg/ml (that refers to 156.25 μg/cm^2^) for 24 or 72 h. Alternatively, viability was assessed after exposing cells to PM samples covering plates at the concentrations up to 625 μg/cm^2^ for 24 h.

### Evaluation of Reactive Oxygen Species Generation by A549 Cells

#### Total Reactive Oxygen Species Production

To evaluate the total ROS production induced in cells after incubation with non-toxic concentrations of PM, the cyto-ID Hypoxia/Oxidative stress detection kit (Enzo Life Sciences, USA) was used according to the manufacturer's protocols. A549 cells were seeded with a density of 2 × 10^4^ cells per cm^2^. A day later, the SRM or ΔC-SRM samples at different concentrations suspended in medium without serum were added and incubated in the dark for 24 h. Then, cells were washed with PBS, treated with trypsin, and analyzed by BD FACSVerse cytometer. As a positive control, pyocyanin (300 μM) was used. The level of the oxidative stress was determined as a percentage of the ROS-positive cells of the whole-cell population determined by pyocyanin control. Alternatively, mean fluorescent intensity of the stained treated cells was evaluated.

To mimic the chronic exposure of A549 cells to PM samples, cells were cultured for 18 days. Firstly, cells were seeded with a density of 2 × 10^4^ cells per cm^2^ on a six-well-plate. Next day, the SRM or ΔC-SRM samples at different concentrations suspended in medium with 2% FBS were added. Cells were incubated with pollutants for 3 days and were reseeded in 1:5 ratio to new plates. The freshly prepared suspensions of PM were added. Cells were incubated in total for 18 days and, every 3 days, cells were reseeded and new suspensions of PM were added. On the 18th day of incubation, A549 cells were washed with PBS, stained with cyto-ID Hypoxia/Oxidative stress detection kit according to the manufacturer's protocols, treated with trypsin, and analyzed by BD FACSVerse cytometer.

To evaluate the influence of PM on the oxidative stress induced by other stimuli, A549 cells were pretreated with PM samples. For this purpose, A549 cells were seeded on a six-well-plate with a density of 2 × 10^4^ cells per cm^2^ a day before. The suspension of SRM or ΔC-SRM samples at different concentrations in medium without serum was added. After 24 h of incubation, cells were washed with PBS and pyocyanin (300 μM) was used for 30 min to induce oxidative stress. A549 cells were then stained with cyto-ID Hypoxia/Oxidative stress detection kit according to the manufacturer's protocols and analyzed by BD FACSVerse cytometer.

#### 2′,7′-Dichlorodihydrofluorescein Diacetate

2′,7′-Dichlorodihydrofluorescein diacetate (DCF-DA) (Sigma-Aldrich) was used to assess the general ROS production by cells exposed to PM samples at non-toxic concentrations. Cells were stained with DCF-DA (20 μM) for 45 min at 37°C. After the staining, the ROS indicator was washed out with PBS, and fluorescence of cells was quantified by a Tecan Infinite 200 plate reader at 535 nm using 485 nm as an excitation wavelength. Control experiments with identical settings but without cells were made to determine the influence of PM on the dye.

#### Qualitative Analysis of the Produced Reactive Oxygen Species

Various fluorescent probes, specific to the selected ROS, were used to determine the type of ROS produced by cells after treatment with SRM or ΔC-SRM samples. Each probe was applied for cells exposed to PM-covered surfaces at two non-toxic concentrations, 50 and 100 μg/cm^2^. After the staining, the ROS indicators were washed twice with PBS, and fluorescence of cells was quantified by a Tecan Infinite 200 plate reader. Controls without cells were made to avoid false-positive results and verify the influence of PM on the used fluorescent probes.

Singlet oxygen sensor green (SOSG, 5 μM, 20 min; ThermoFisher Scientific) was used to assess the production of singlet oxygen in cells. Aminophenyl fluorescein (APF, 5 μM, 30 min, ENZO) was used to evaluate the production of hydroxyl radical, hypochlorite, or peroxynitrite. Dihydrorhodamine 123 (DhR123, 10 μM, 20 min, AAT Bioquest) was used to estimate the production of mainly hydrogen peroxide; however, it can be used to detect additionally hypochlorite, peroxynitrite, or cytochrome c. Hydroethidium (HE, 10 μM, 20 min, AAT Bioquest) was used to check the production of superoxide anion radical. MitoSox Red (5 μM, 10 min, ThermoFisher Scientific) was applied to evaluate the production of superoxide anion radical in mitochondria. Staining cells with probes was performed at 37°C. Fluorescence intensity of cells was measured at 535 nm using 485 nm as an excitation wavelength for SOSG/APF and at 529 nm using 507 nm as an excitation wavelength for DhR123. For HE evaluation, fluorescence intensity of cells was quantified at 605 nm using 520 nm as an excitation wavelength, while 510/595 nm were used as excitation/emission wavelengths for MitoSox Red quantification.

### Lipid Peroxidation

Lipid peroxidation was assessed using C_11_-BODIPY^581/591^-Lipid Peroxidation Sensor (ThermoFisher Scientific). A549 cells were seeded on PM-covered surfaces at two concentrations, 50 and 100 μg/cm^2^, 24 h before the experiment. After that, cells were stained with C_11_-BODIPY^581/591^ (1 μM) for 30 min in the dark at 37°C. The fluorescence intensity of cells was measured at 484/510 nm (green) and 581/610 nm (red)—the excitation/emission wavelengths.

### Mitochondrial Membrane Potential

Mitochondrial membrane potential (ΔΨm) was evaluated using JC-1 probe (AAT Bioquest). The JC-1 probe is a lipophilic cationic dye that exhibits potential-dependent accumulation in the mitochondria. At low ΔΨm, the probe exists as a monomer that emits green fluorescence. At higher ΔΨm, the concentration of dye increases and the probe forms J-aggregates that lead to a shift in fluorescence emission from green to red. A change from red to green fluorescence reflects a decrease in ΔΨm. A549 cells were exposed to PM-covered plates at two concentrations, 50 and 100 μg/cm^2^. After 24 h of treatment, cells were stained with JC-1 (10 μM) for 30 min in the dark at 37°C. The fluorescence intensity of cells was measured at 525 and 590 nm using 490 nm as an excitation wavelength.

### Cytosolic Calcium Homeostasis

Cytosolic calcium concentration was measured using Fluo-8 AM probe (AAT Bioquest). A549 cells were exposed to PM-covered plates at two concentrations, 50 and 100 μg/cm^2^. After 24 h of treatment, cells were stained with Fluo-8 AM (4 μM) for 30 min in the dark at 37°C. The fluorescence intensity of cells was measured at 525 nm using 490 nm as an excitation wavelength.

### The Mechanism of Cellular Death

The mechanism of cellular death was evaluated using Annexin V-fluorescein isothiocyanate (FITC)/propidium iodide (PI) (ThermoFisher Scientific) assay. The phosphatidylserine of the cytoplasmic membrane at the early stage of apoptosis flipped on the outer surface of cell membrane and in the presence of calcium ions is bound with Annexin V. PI was used to assess the necrotic population of cells. A549 cells were seeded on PM-covered plates with a density of 4 × 10^4^ cells per cm^2^, and 24 h after the seeding, the test was performed. A549 cells were stained with Annexin V-FITC for 10 min in the dark and then with PI (0.5 μM) for 5 min. Cells were analyzed by a BD FACSVerse cytometer.

### Caspase Activity

Activation of caspases 3/7 was examined using CellEvent Caspase-3/7 Green Detection Reagent (ThermoFisher Scientific). A549 cells were seeded on PM-covered plates with a density of 4 × 10^4^ cells per cm^2^. Twenty-four hours later, cells were stained with CellEvent Caspase-3/7 Green Detection Reagent. The fluorescence intensity of cells was analyzed using a BD FACSVerse cytometer.

### Statistical Analysis

For *in vitro* experiments, all data were expressed as the mean ± standard error of the mean (SEM). All the experiments were performed in triplicate and repeated at least three times. Significant differences among groups were determined using *t*-test or a one-way analysis of variance (ANOVA) using OriginPro 2018 software. Probabilities of *p* < 0.05 were considered as statistically significant. The following notification is used ^*^p < 0.05, ^**^p < 0.001.

## Results and Discussion

For the evaluation of biological effect of PM with and without organic components *in vitro*, A549 cell line was used. This line was chosen as a simple model of lung epithelial cell line as has been routinely used for this type of studies (Billet et al., 2007; Mehta et al., 2008; Wang et al., 2013; Huang et al., 2014, 2015; Lee et al., 2014; Lu et al., 2014; Schilirò et al., 2015; Kim et al., 2018; Li et al., 2020). SRM 1648a sample (SRM) supplied by the National Institute of Standards and Technology, USA, was used as a standardized sample for urban air pollutants. Sample without organic contaminations (ΔC-SRM) was prepared using low-temperature plasma treatment during which the organic carbon content decreased from 14 to 1.8%, as determined by both elementary analysis and TOC measurements. Neither significant changes in the morphology nor aggregation of particles due to plasma treatment was observed (Mikrut et al., 2018). Two different exposure protocols were used to fully assess the influence of PM on biological parameters of A549 cells: i) suspension of PM in cell culture medium (exposure method 1) and ii) PM-covered plates (exposure method 2).

### Cell Viability

The viability of the A549 cells after exposure to SRM and ΔC-SRM samples was assessed using MTT or resazurin tests. It was determined that after 24 h of incubation with either SRM or ΔC-SRM samples suspended in medium, the viability of A549 cells decreased significantly for the concentrations higher than 100 μg/ml (Supplementary Figure 1A). These results are in agreement with other reports (Holian et al., 1998; Gawda et al., 2018). No difference in toxicity between SRM and ΔC-SRM was observed for 24 h of incubation, while after 72 h, SRM sample appeared to be more toxic (Supplementary Figure 1B). Prolonging the incubation time in case of ΔC-SRM sample did not induce additional toxicity. Serum proteins may have substantial impact on the toxicity of numerous compounds by either reducing the toxicity due to the lower accumulation of compounds or increasing the toxicity by facilitating the uptake (Mazuryk et al., 2014). Therefore, the influence of fetal bovine serum (FBS) presence in the incubation medium was studied by performing the viability assay additionally in the presence of 2 or 10% FBS. It was determined that FBS has a negligible impact on the toxicity of SRM sample (results are not shown).

The decrease in viability of the A549 cells after the incubation with SRM or ΔC-SRM samples may not be solemnly caused by chemical nature of PM interaction with cells. Additional factors related to the physical contact and mechanical disturbance of cells by PM sample should be considered (Supplementary Figure 2). PM applied directly on cells, covering them that can efficiently reduce the amount of nutrients and generate mechanical damage, which in consequence may induce cell death.

To eliminate this effect, plates were covered with PM samples at different concentrations and then used for cell seeding (see experimental part). Additionally, some plates covered with PM samples were coated with collagen or fibronectin prior to seeding of cells. Plates prepared in such a way gave cells the ability to interact with PM without limiting a proficient collection of nutrients from cell medium. Covering plates with PM samples and coating them with adhesion proteins next significantly reduced the toxicity of SRM or ΔC-SRM samples (Supplementary Figure 3). No toxicity of PM samples was observed in cells seeded on uncoated plates or plates coated with collagen, while slight toxicity (up to 80% viability) was observed on the surface coated with fibronectin. PM covering technique allowed the use of 20 times more concentrated PM samples (100 μg/ml = 31.25 μg/cm^2^) as a nontoxic dosage.

Attention should be paid to morphological changes of A549 cells upon contact with PM (Supplementary Figure 4). Flow cytometry results revealed only a slight decrease in cell size (FSC) while cell granularity parameter (SSC) increased. This indicates that PM was collected by cells. Plasma-treated and untreated PM samples induced a similar effect on the SSC parameter.

### Evaluation of Reactive Oxygen Species

The generation of ROS in A549 cells upon interaction with PM samples was explored using two different methods of contact between cells and PM. The samples were applied either in the form of suspension in medium or as a layer covering the plate.

**Figure 1:**
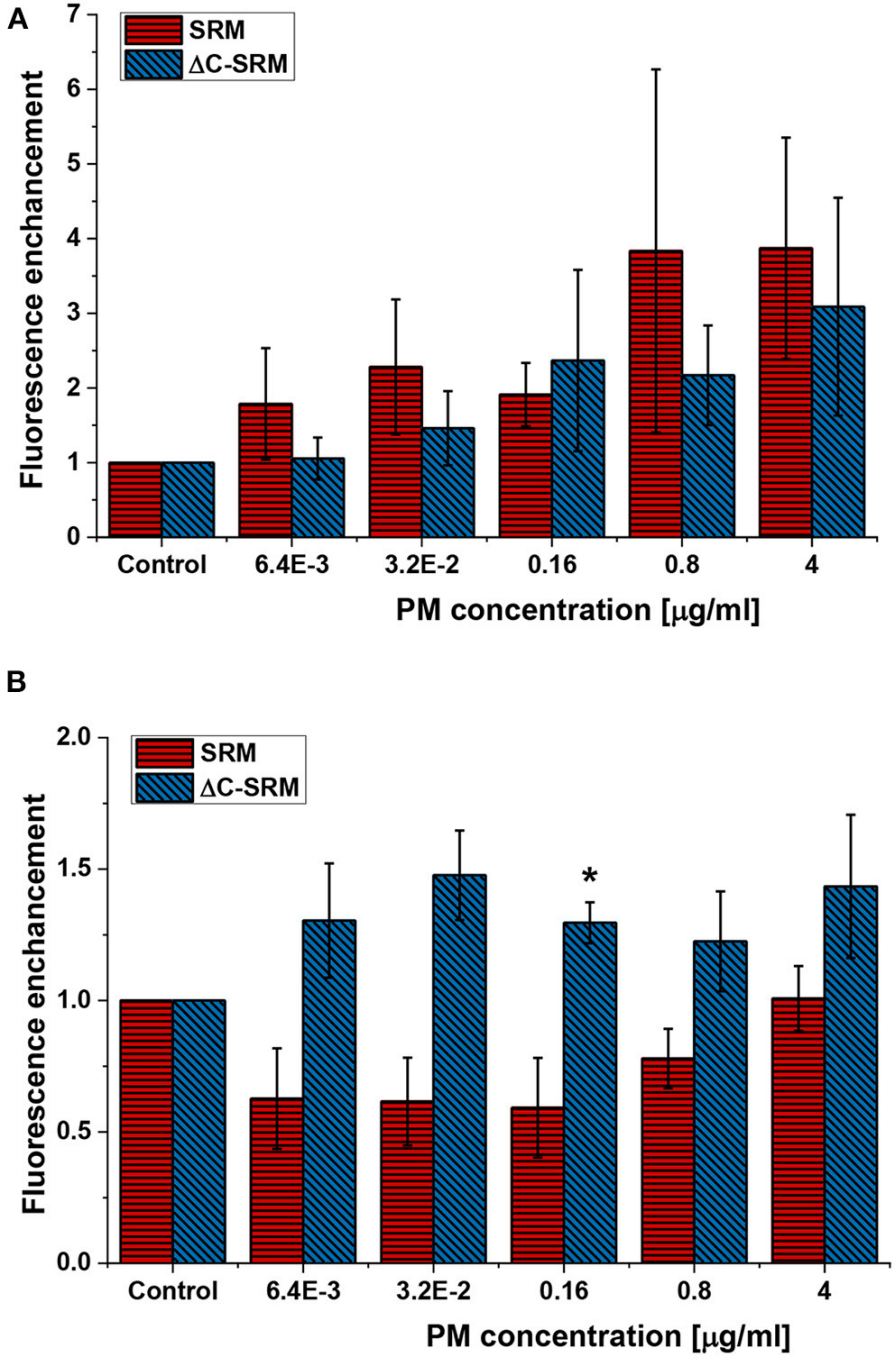
The reactive oxygen species (ROS) production in A549 cells after their exposure to suspensions of SRM or ΔC-SRM for 24 h **(A)** and 18 days **(B)** was evaluated using cyto-ID Hypoxia/Oxidative stress detection kit (exposure method 1), *p < 0.05.

ROS production was assessed using small non-toxic concentrations of PM to avoid false-positive results caused by cells' response to toxic effect of PM or PM interference with fluorescent probes. Total ROS production in A549 cells after different incubation times was evaluated using cyto-ID Hypoxia/Oxidative stress detection kit. One hour of incubation with SRM suspension was not a sufficient period of time to produce a significant amount of ROS by cells (Supplementary Figure 5). However, treatment with suspension of inorganic part of pollutants (ΔC-SRM sample) was able to cause a detectable increase in the amount of ROS produced by A549 cells even after 1 h of incubation (Supplementary Figure 5). Twenty-four hours of cell exposure to PM suspensions resulted in an increase in ROS-positive cell population for both applied PM samples, SRM and ΔC-SRM (Figure 1A). The SRM sample induced a stronger effect, confirming relevance of organic pollutants in oxidative stress induction. Semi-chronic exposure (18 days) to PM suspensions caused a different effect (Figure 1B). Incubation of A549 cells with SRM sample did not result in an increase in ROS production, indicating an existence of a compensatory mechanism toward constant small exposure to pollutants. Some of the organic pollutants have ROS-scavenging properties, and they may act as antioxidants. In contrast, ΔC-SRM induced a significant rise in the amount of generated ROS in cells, fairly independent of the used concentration of the sample, suggesting a catalytic mechanism of ROS production. High content of metals such as Fe, Cu, and Mn in ΔC-SRM can participate in catalytic production of ROS.

**Figure 2:**
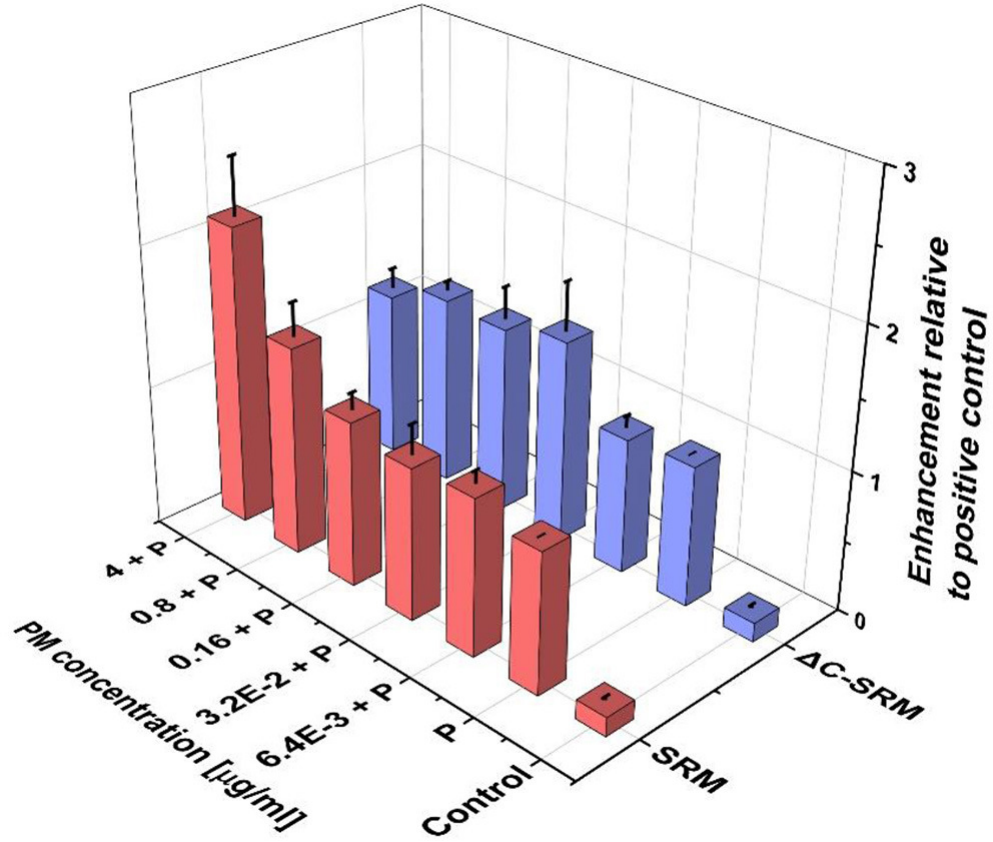
The reactive oxygen species (ROS) production in A549 cells pretreated with particulate matter (PM) suspensions for 24 h and subsequently exposed to pyocyanin (P) was evaluated using cyto-ID Hypoxia/Oxidative stress detection kit (exposure method 1).

To further evaluate the influence of PM on oxidative stress induction, the production of ROS in cells pretreated with PM suspensions (SRM or ΔC-SRM) and exposed to a known ROS inducer was studied. As a ROS inducer, pyocyanin was used, a toxin produced by *Pseudomonas aeruginosa* (Ando and Yonamoto, 2015; Winter and Zychlinsky, 2018). Pretreatment of cells with PM suspensions for 24 h before exposing them to pyocyanin resulted in a higher response to the used stimuli (Figure 2). Pretreatment with SRM resulted in a strong dose-dependent increase in ROS production upon exposure to pyocyanin, while pretreatment with ΔC-SRM induced a similar effect but independent of concentration. The results are in agreement with literature data, which showed that exposure of murine macrophages to suspension containing low concentrations of PM may prime cells to a hyper-inflammatory response upon contact with the second stimulus (Gawda et al., 2018).

In numerous publications, the production of ROS was evaluated using DCF-DA assay (Wilson et al., 2002; Baulig et al., 2004, 2009; Andersson et al., 2009; Lee et al., 2014, 2016; Rodríguez-Cotto et al., 2015; Ahlberg et al., 2016). DCF-DA is a cellular probe that reacts with numerous ROS to produce fluorescent product and is often used to estimate the general ROS production. The level of oxidative stress in A549 cells was measured by this probe after 24- or 72-h incubation with PM suspension. No significant ROS production in A549 cells using DCF-DA probe was observed after 24 h of incubation for each of the samples (Supplementary Figure 6A). Prolonging incubation time up to 72 h resulted only in a slight increase in the level of oxidative stress (Supplementary Figure 6B). Such discrepancy in the results between two tests used for the evaluation of ROS production can be explained by different sensitivities of the probes, since DCF-DA is mostly sensitive toward H_2_O_2_, while cyto-ID Hypoxia/Oxidative stress detection kit detects general ROS production (according to the manufacturer's protocol). The amount of ROS generated by suspension of PM may not be high enough to be detected by DCF-DA probe. Further increase in PM concentration was not possible due to its toxicity (Supplementary Figure 1). Additionally, PM concentration higher than 20 μg/ml (for SRM sample) and 10 μg/ml (for ΔC-SRM sample) interfered with DCF-DA assay, causing its oxidation.

**Figure 3:**
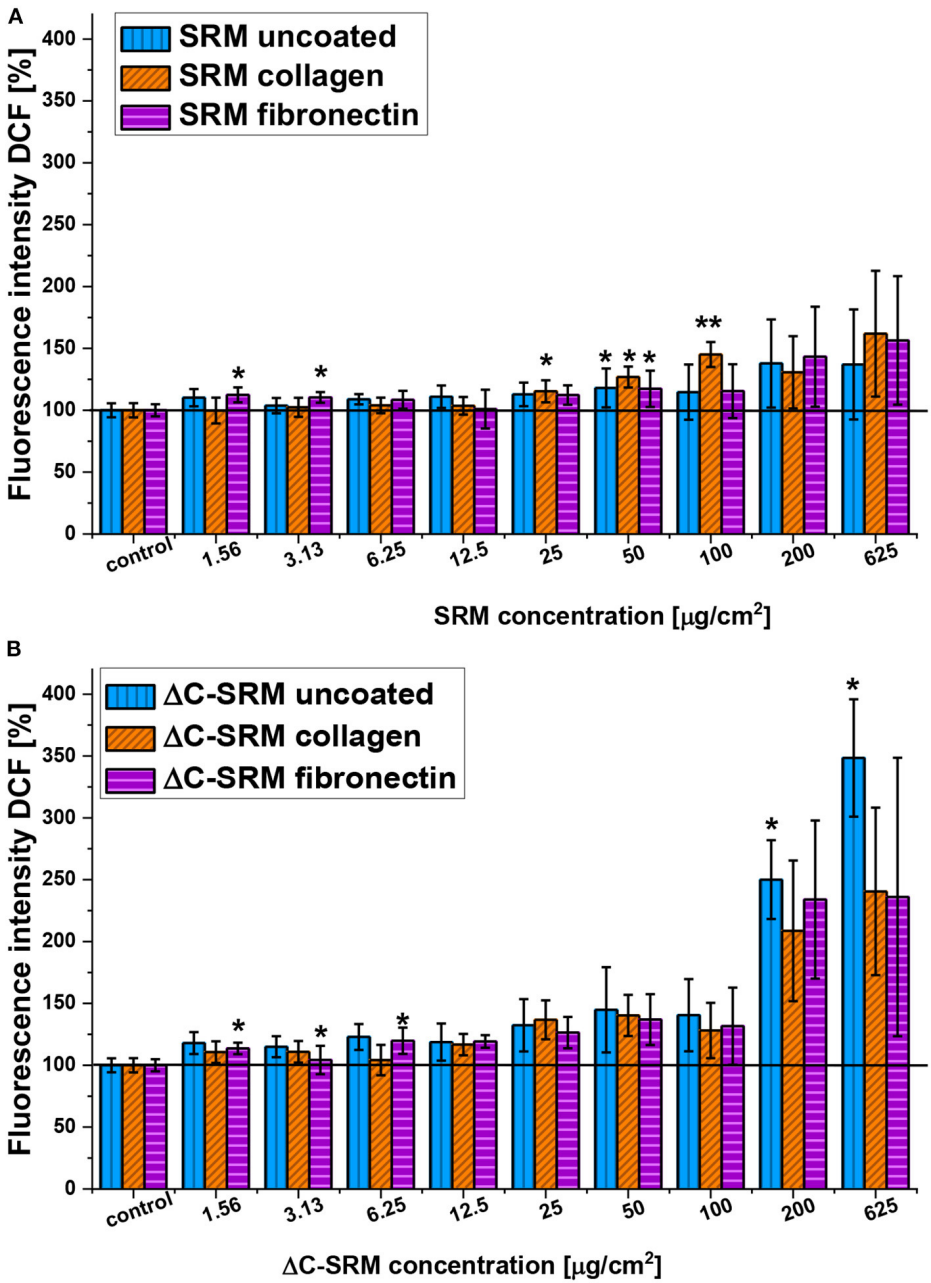
The reactive oxygen species (ROS) production in A549 cells after 24-h incubation with particulate matter (PM) suspensions of different concentrations covering plates either uncoated or coated with fibronectin/collagen measured using 2,7-dichlorodihydrofluorescein diacetate (**A**, SRM; **B**, ΔC-SRM) (exposure method 2),*p < 0.05, **p < 0.001.

To overcome the mentioned problems for further studies, the PM covering technique was used. Covering plates with PM samples and coating them with adhesion proteins resulted in a significant decrease of toxicity of air pollutants (Supplementary Figure 3); therefore, the higher amount of PM samples, still in nontoxic range of concentration, can be used in the evaluation of ROS production in A549 cells by using DCF-DA assay. The production of ROS in A549 cells seeded on the surface covered with PM samples and coated with either fibronectin or collagen is shown in Figure 3. Both SRM and ΔC-SRM samples induce oxidative stress in A549 cells. ΔC-SRM samples were more efficient as ROS inducers. They caused ca. twice higher ROS production than SRM samples when cells were treated with a high concentration of PM (>200 μg/cm^2^). SRM samples started to induce significant oxidative stress in the concentration higher than 50 μg/cm^2^, while ΔC-SRM samples caused a similar level of ROS production with a concentration as small as 1.6 μg/cm^2^.

To thoroughly evaluate the role of inorganic fraction of PM samples in ROS formation in treated cells, the mass reduction of plasma-treated sample was taken into account. During cold plasma treatment, ca. 33% decrease in mass of the sample was observed, so the same mass of ΔC-SRM in comparison to SRM contained much more inorganic particles. To compensate for this effect, the results of DCF-DA evaluation of ROS formation were recalculated, and ROS formation effect of SRM was compared to 67% of ROS generation induced by ΔC-SRM (Supplementary Figure 7). At small and moderate concentrations of PM samples (up to 100 μg/cm^2^), SRM induced a greater increase in ROS production in A549 cells. However, higher concentrations (>200 μg/cm^2^) resulted in a similar level of oxidative stress induction by both SRM and ΔC-SRM samples. These results confirmed that at small to moderate concentrations of PM, the organic fraction plays an important role in an oxidative stress formation; however, it is not exclusively responsible for it. At higher concentrations, catalytic activity of inorganic particles became more evident and ΔC-SRM samples induced similar production of ROS as SRM samples.

### Qualitative Analysis of the Produced Reactive Oxygen Species

**Figure 4:**
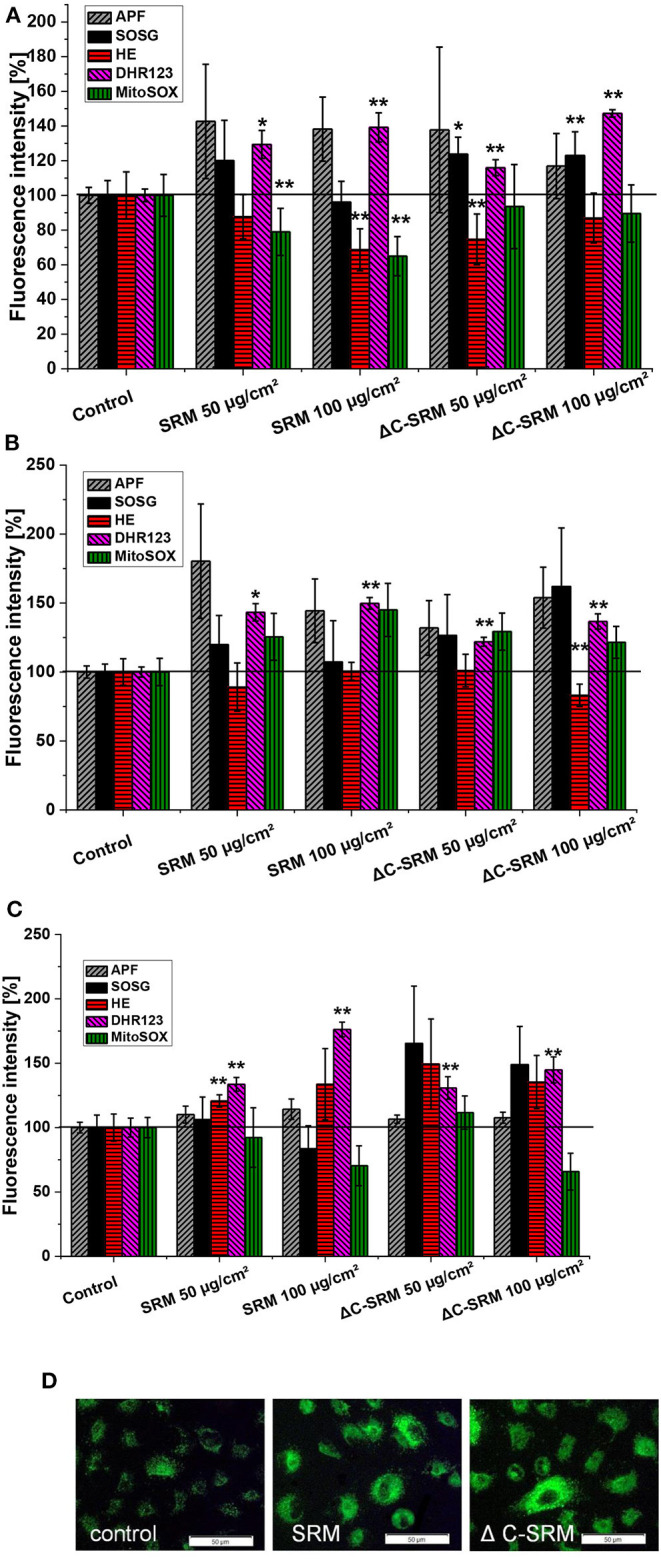
The level of reactive oxygen species (ROS), measured by selective fluorescent probes, induced in A549 cells after their 24-h incubation on particulate matter (PM)-covered plates either uncoated **(A)** or coated with collagen **(B)** and fibronectin **(C)** (exposure method 2). SOSG, Singlet Oxygen Sensor Green (^1^O_2_); APF, aminophenyl fluorescein (**·**OH, ONOO^−^, HOCl); DhR123, dihydrorhodamine 123 (H_2_O_2_, ONOO^−^, HOCl, cytochrome c); HE, hydroethidine (${\text{O}}_{2}^{\cdot -}$); MitoSox™ (${\text{O}}_{2}^{\cdot -}$ in mitochondria). **(D)** Representative images of ROS production induced in A549 cells on PM-covered plates (100 μg/cm^2^) measured by DhR123. Scale bar is 50 μm. *p < 0.05, **p < 0.001.

To determine a profile of the produced ROS, the selective fluorescent probes, described in detail in the experimental part, were used. A549 cells were grown on plates covered with PM at concentrations 50 and 100 μg/cm^2^ to avoid possible interaction of PM samples with fluorescent probes. Incubation of A549 cells on SRM-covered plates resulted in increased fluorescent signals from APF, DhR123, HE (only on fibronectin-coated plate), and MitoSox (only on collagen-coated plate) indicators (Figure 4). This indicated that majority of ROS produced by A549 upon contact with SRM are hydrogen peroxide (H_2_O_2_), hydroxyl radical (^**·**^OH), and superoxide anion radical (${\text{O}}_{2}^{\cdot -}$). Plasma-treated air pollutant (ΔC-SRM) sample upon contact with A549 cells additionally to already mentioned ROS induced the production of singlet oxygen, as was manifested by the increased fluorescent signal from the SOSG probe (Figure 4). The lack of singlet oxygen production by cells treated with SRM samples can be a consequence of an antioxidant potential of organic part of PM sample. Singlet oxygen can be quenched by and/or reacts with many organic molecules (DeRosa and Crutchley, 2002).

### Lipid Peroxidation

**Figure 5:**
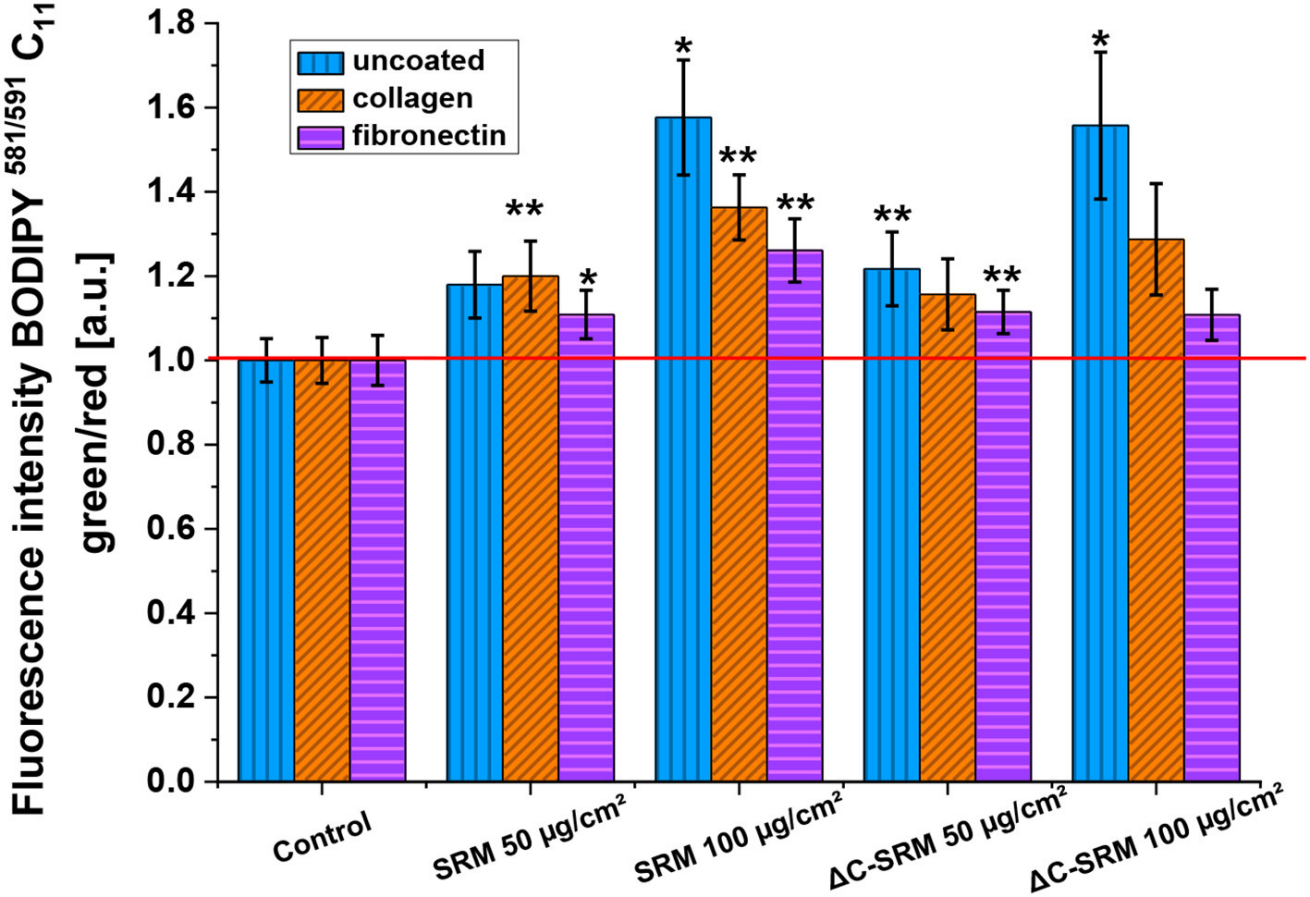
The lipid peroxidation in A549 cells after 24-h incubation with different concentrations of particulate matter (PM)-covered plates either uncoated or coated with fibronectin/collagen measured using C_11_-BODIPY^581/591^ probe (exposure method 2), *p < 0.05, **p < 0.01.

Lipid peroxidation was assessed using C_11_-BODIPY^581/591^-Lipid Peroxidation Sensor (Figure 5). This liposoluble probe upon oxidation with lipid peroxyl radicals (ROO^**·**^) alters its fluorescence properties, shifting emission intensity to shorter wavelengths, from red to green fluorescence. Incubation of A549 cells on the PM-covered surfaces resulted in increased green-to-red fluorescence ratio of C_11_-BODIPY^581/591^ probe, displaying the occurrence of lipid peroxidation. The observed effect was similar for SRM and ΔC-SRM samples, which can indicate that the organic part of PM has a negligible effect on lipid peroxidation.

### Mitochondrial Membrane Potential

**Figure 6:**
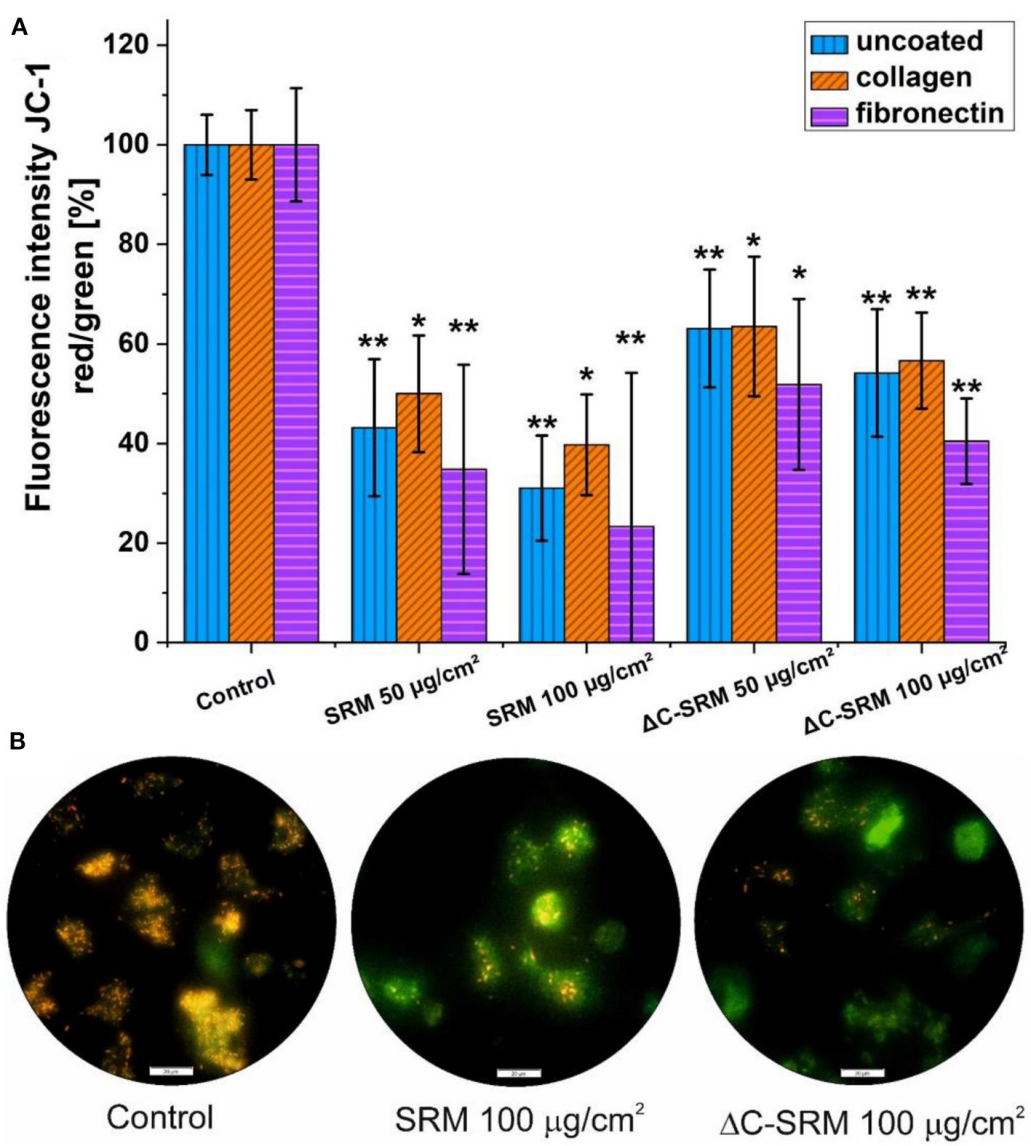
**(A)** The changes in mitochondrial membrane potential (ΔΨm) in A549 cells after 24-h incubation on particulate matter (PM)-covered plates either uncoated or coated with fibronectin/collagen measured using JC-1 probe (exposure method 2). **(B)** Representative images of depolarization of ΔΨm of A549 cells induced by PM. Scale bar is 20 μm; *p < 0.05, **p < 0.001.

To evaluate ΔΨm, JC-1 probe was used (Figure 6). The shift in mitochondrial potential was represented as a change in red/green fluorescence ratio. Both untreated and plasma-treated PM samples caused the decrease in red/green fluorescence intensity ratio, indicating depolarization of the mitochondrial membrane. The effect was concentration dependent and was slightly stronger after exposure cells to SRM samples.

### Intracellular Calcium Concentration

Calcium ion is one of the most important signal transducers in cells, involved in numerous physiological and pathological processes including proliferation, differentiation, and cellular motility. Both calcium spatial localization and magnitude might determine the cell's faith. It is well-known that various metals can modify intracellular Ca^2+^ signaling (MacNee and Donaldson, 2003). Fluo-8 AM probe was used to determine the changes in intracellular calcium concentration in A549 cells after 24-h incubation on the PM-covered plates uncoated or coated with fibronectin/collagen (Supplementary Figure 8). Incubation of A549 cells on PM samples resulted in the decrease in Fluo-8 AM probe fluorescence, indicating the disruption of calcium homeostasis and the decrease in cytosolic [Ca^2+^]. The results are more pronounced for cells seeded on uncoated plates than for one coated with adhesion proteins.

### Mechanism of Cellular Death

**Figure 7:**
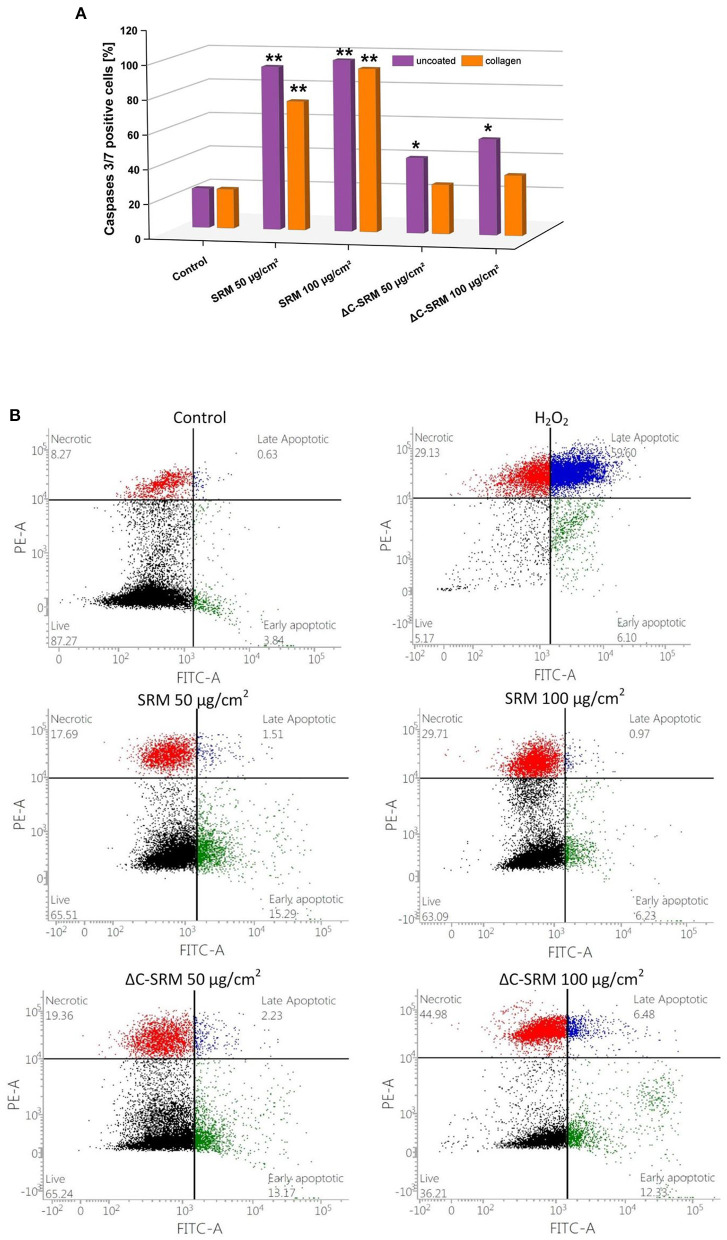
**(A)** The percentage of caspase 3/7-positive cells after 24-h incubation with different concentrations of particulate matter (PM)-covered plates either uncoated or coated with fibronectin/collagen (exposure method 2). **(B)** The percentage of living, necrotic, and apoptotic A549 cells after 24-h incubation on PM-covered plates (exposure method 2). *p < 0.05, **p < 0.001.

To investigate the mechanism of cellular death, the activity of executioner caspases 3/7 was examined. These enzymes are activated during regulated cellular death (extrinsic or intrinsic apoptosis) and are responsible for many morphological and biochemical changes such as DNA fragmentation, phosphatidylserine exposure, and formation of apoptotic bodies (Galluzzi et al., 2018). The percentage of caspase 3/7-positive cells after 24-h incubation with different concentrations of PM-covered plates is shown in Figure 7A. In A549 cells after exposure to PM samples, the percentage of caspase 3/7-positive cells significantly increased. SRM sample caused activation of executioner caspases in the whole population of the treated cells, while ΔC-SRM sample only in around 30%. The results indicate that apoptosis is a main mechanism of cellular death for SRM-treated cells, while exposure to ΔC-SRM can induce some additional caspase-independent cell death mechanisms.

To further investigate the mechanism of cellular death, morphological signs of apoptosis and necrosis were examined by Annexin V-FITC/PI assay. This assay allows for determination of a potential loss of cellular integrity (during necrosis) and measures transition of phosphatidylserine (PS) on the outer surface of the cell membrane (during apoptosis). The percentage of living, necrotic, and apoptotic A549 cells after 24-h incubation with different concentrations of PM-covered plates is shown in Figure 7B. The presence of SRM and ΔC-SRM samples increased the population of apoptotic and necrotic cells. While some necrotic fraction of the populations can be explained by end-stage apoptosis (Galluzzi et al., 2018), inorganic PM sample, ΔC-SRM, undoubtedly induced a stronger effect on A549 cells than the SRM sample, producing a significantly higher amount of necrotic cells. The results indicate involvement of necrotic cell death as cells' failed responses to adapt to induced stress. Similar results were obtained for collagen-coated surfaces (results are not shown).

## Critical Discussion of The Results

Several studies tried to estimate the relative contribution of inorganic and organic components to PM-induced toxicity (Gao et al., 2020). Some studies try to do this by multiplying the intrinsic oxidative potential of individual compounds of PM with their ambient concentrations and provide evidence of the importance of either transition metals (Charrier and Anastasio, 2012) or organic components (Verma et al., 2015). Others compared atmospheric particles with different amounts of organic constituents such as PM2.5, carbon black particles, and diesel exhaust particles (DEPs). Baulig et al. concluded that organic compounds presented in DEP (Baulig et al., 2003) or isolated from PM2.5 (Baulig et al., 2009) mainly contributed to the observed biological effects in human bronchial epithelial cells. Our approach relies on the evaluation of inorganic fraction contribution on biological response to airborne PM by removing organic compounds using cold plasma treatment. Such treatment does not induce changes in particle morphology or size; however, we are not able to gain detailed information on the speciation of the PM components.

Due to the cumulative nature of pollution, adverse health effects are often results of chronic exposure to harmful particles, so when cells are exposed to urban airborne PM at quantities expected from the known content of PM in air, the observed changes are at a very low level or negligibly small. Large quantities of PM that have often been used to detect changes in various biological parameters *in vitro* can induce additional mechanical damage, which will falsify the obtained results due to the ability of a dying cell to expose or release molecules that alert other cells. In these studies, special emphasis was placed on the elimination of false-positive results arising from mechanical pressure of PM on cell culture. This was achieved through an inverting cell exposure protocol by covering cell culture plates with PM. Such alternative way of cell treatment allowed us to increase 20 times the used PM concentration without causing unnecessary cell death from the mechanical damage.

Another serious issue in this type of research concerns the oxidation of the fluorescent probes by using PM due to the oxidative potential of both organic and inorganic components of airborne air pollution. To eliminate such a possibility, several control experiments were carried out and carefully analyzed to avoid artificial positive results. The combination of these factors allowed for the selection of an appropriate cell treatment protocol and PM concentrations that make it a possibility to study narrow differences between the tested PM samples.

We are aware that such a model system also has some limitations. One of the most relevant is the inability to assess synergistic and antagonistic interactions between different PM components that could significantly alter their redox properties. This method does not consider the cumulative effect of pollution and can underestimate the influence of fraction due to its low atmospheric concentration. Additionally, bulk PM mass measurements do not accurately represent a complex chemical mixture that is air pollution, which additionally varies both spatially and temporally. *In vitro* studies using lung epithelial cells allowed assessing the effects of dissolved components and components of the studied PM with a size smaller than 2.5 μm. Only such particles are capable of reaching the alveolar epithelium, crossing cell membranes, and directly interacting with cellular structures. SRM predominantly contains particles from 5 to 20 μm, which can lead to underestimation of the observed effects. The negative effects of air pollution on human health is difficult to imitate under laboratory conditions; however, by using thoroughly characterized Standard Reference Materials (available from the National Institute of Standard and Technology), the effect of airborne PM can be studied among different laboratories worldwide.

## Conclusions

Air pollution is now recognized as one of the main health risks in the developed world. The presented work is focused on the comparison of biological effects *in vitro* for the organic and inorganic fractions of PM. To achieve the goal, the influence of intact standardized urban PM (SRM) and plasma-treated SRM containing predominantly inorganic fraction of PM (ΔC-SRM) on various biological parameters of A549 lung epithelial cells was directly compared following the newly elaborated experimental protocols.

The obtained results indicate a significant influence of the inorganic part of PM on oxidative stress generation. Both organic and inorganic fractions of PM are involved in the induction of ROS production by cells. In the case of small and moderate concentrations of PM and shorter incubation time (24 h), the organic fraction of PM plays a pivotal role in oxidative stress induction. The significance of the inorganic fraction is enhanced with increasing PM concentrations and prolonging incubation time (up to 18 days), mimicking in this way chronic exposure. Covering plates with PM allowed the use of higher concentrations of air pollutants in the studied suspensions without observing the toxic effect of the particles. At high concentrations, treatment with ΔC-SRM demonstrated a similar effect on ROS production as SRM, and the catalytic activity of inorganic particles became more apparent.

Both air pollutant samples decreased the viability of A549 cells by decreasing the ΔΨm and disrupting calcium homeostasis. Despite the SRM sample being slightly more toxic toward A549 cells, the inorganic fraction (ΔC-SRM) appeared to be more dangerous due to its superior ability to induce necrosis in the studied cell line. The biological alterations observed *in vitro* might contribute to lung inflammation and increased probability of pulmonary diseases.

Detected changes in the observed biological effects of SRM and ΔC-SRM might be related to differences in the ROS profile induced in cells. Singlet oxygen production was observed only after treatment of cells with ΔC-SRM. Hydrogen peroxide, hydroxyl radical, and superoxide anion radical are the most abundant ROS produced after exposure of A549 cells to SRM, while ΔC-SRM preferably induced the production of H_2_O_2_, singlet oxygen, and hydroxyl radical.

The obtained findings are of importance for our future understanding how different organic and inorganic components of PM contribute to the adverse health effects of PM and could help to develop a strategy to identify the most harmful components. The proposed novel research protocol is crucial for accurate evaluation of the biological effects of PM *in vitro* without cell exposure to additional stressful factors such as physical disturbance.

## Data Availability Statement

The raw data supporting the conclusions of this article will be made available by the authors, without undue reservation.

## Author Contributions

All authors listed have made substantial, direct and intellectual contribution to the work, and approved it for publication.

## Conflict of Interest

The authors declare that the research was conducted in the absence of any commercial or financial relationships that could be construed as a potential conflict of interest.
