# Supplementary material for: Variations in Reactive Oxygen Species Generation by Urban Airborne Particulate Matter in Lung Epithelial Cells—Impact of Inorganic Fraction
Source: Front Chem. 2020 Dec 17;8:581752. doi: 10.3389/fchem.2020.581752 (PMC7773840; doi:10.3389/fchem.2020.581752)
Supplement: Supplementary file 1 [file Data_Sheet_1.PDF]

Variations in reactive oxygen species generation by urban airborne particulate matter in lung epithelial cells – impact of inorganic fraction.

Olga Mazuryk*, Grażyna Stochel, Małgorzata Brindell

Faculty of Chemistry, Jagiellonian University, ul. Gronostajowa 2, 30-387, Kraków, Poland

Supplementary material

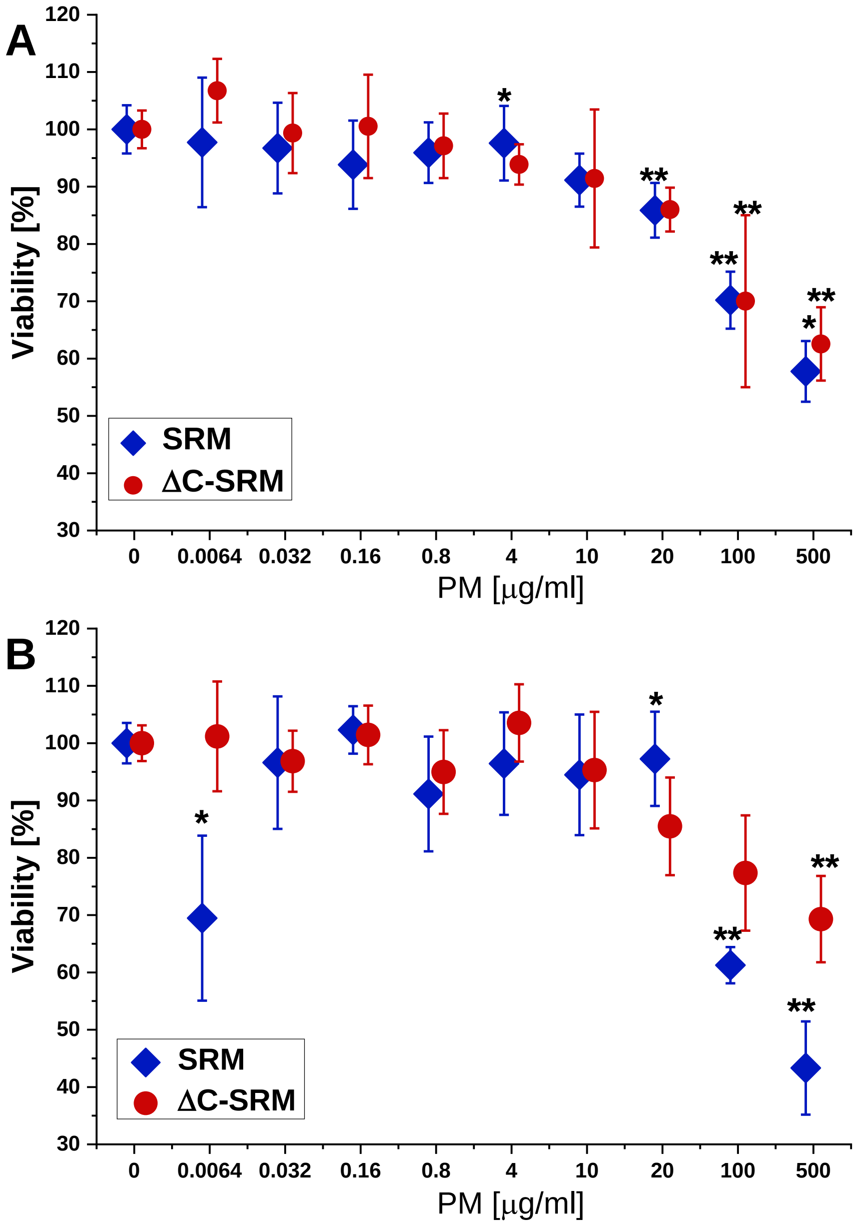

Fig. S1. The viability of A549 cells after exposure to different concentration of SRM or ΔC-SRM samples suspended in medium without serum for 24 h (A) and 72 h (B) measured by MTT test. The results are representative of at least three independent experiments performed in triplicate and are expressed as mean ± SEM (error bars) of replicates* $p < 0.05$, ** $p < 0.01$.

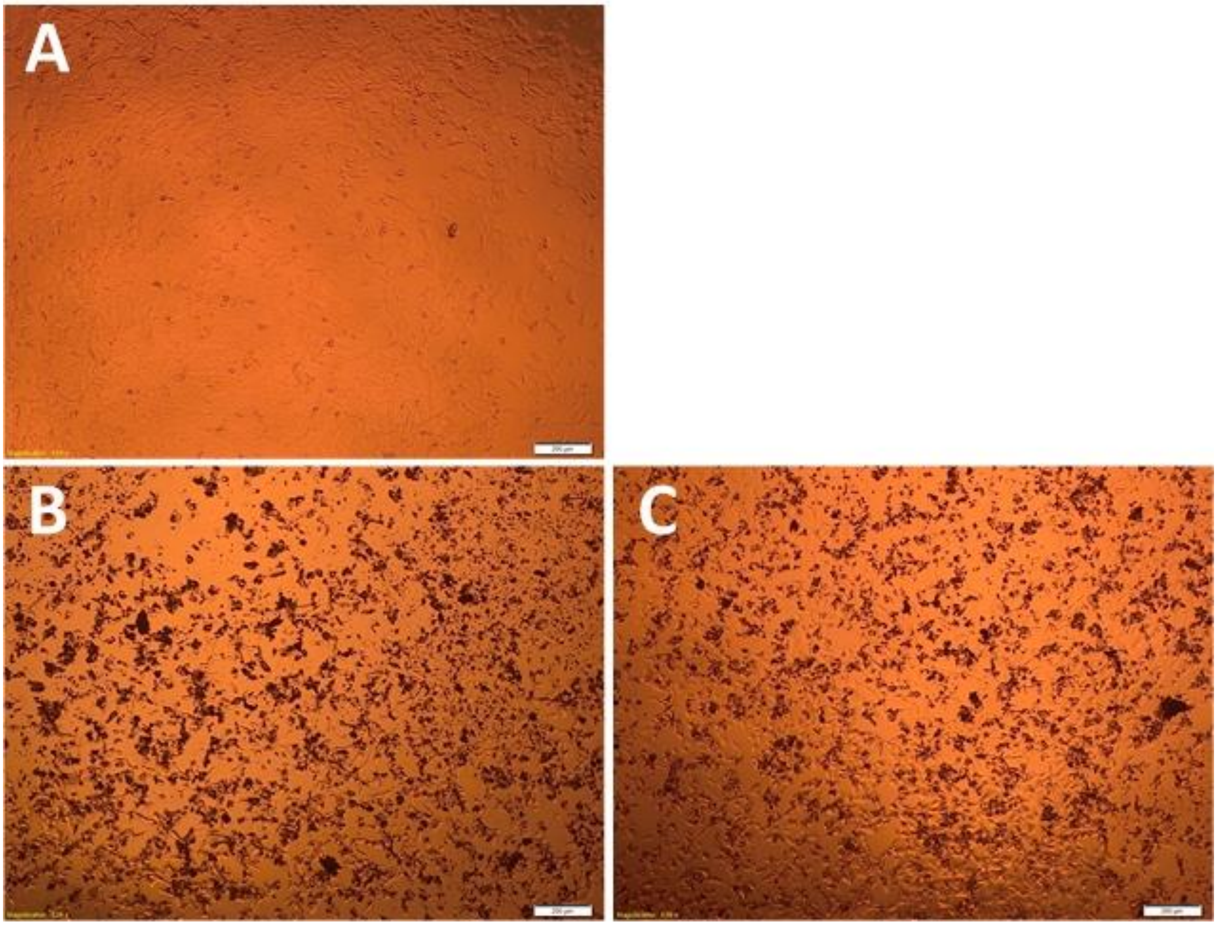

Fig. S2. Representative images of A549 cells after incubation with SRM 100 μg/ml (B) and ΔC-SRM 100 μg/ml (C) samples for 24 h, where (A) – control cells. Scale bars 200 μm.

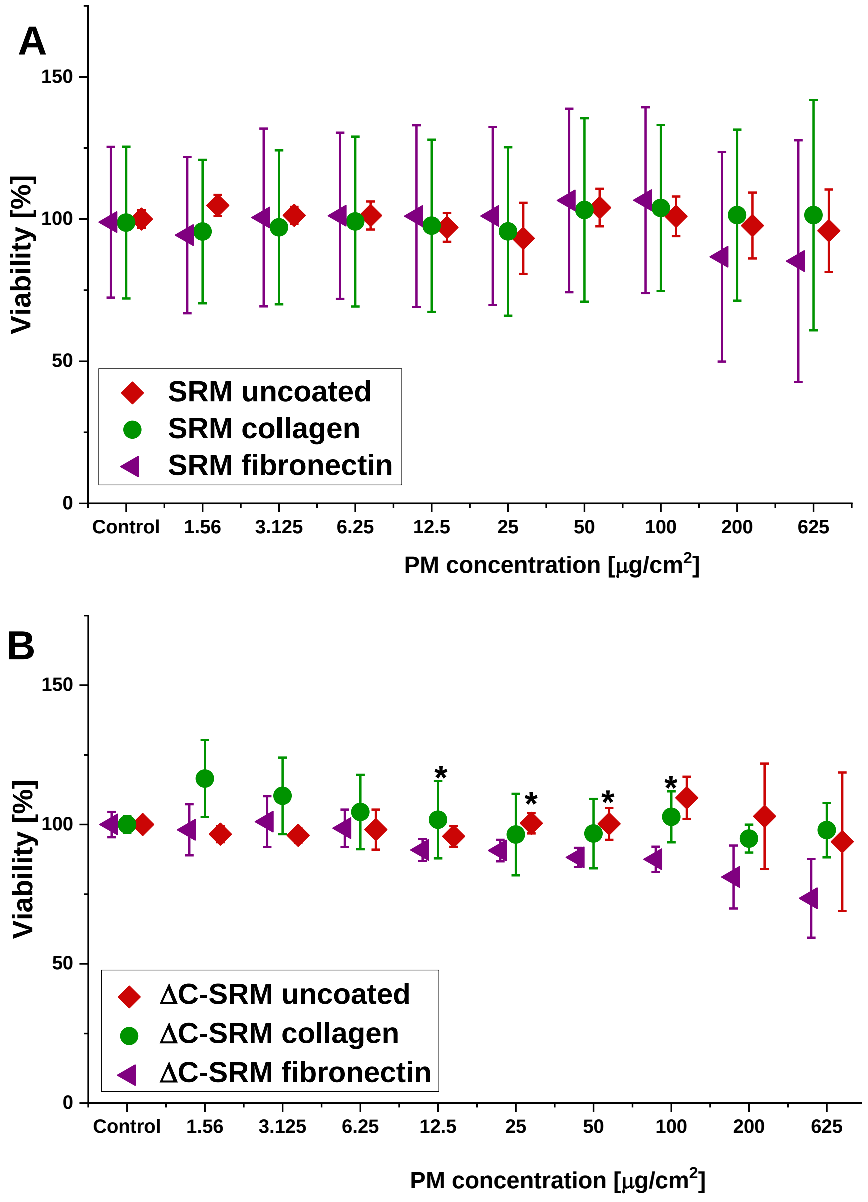

Fig. S3. The viability of A549 cells after 24 h exposure to different concentrations of PM samples (A – SRM, B – ΔC-SRM) covered uncoated/coated with fibronectin or collagen surfaces measured by resazurin test. The results are representative of at least three independent experiments performed in triplicate and are expressed as mean ± SEM (error bars) of replicates, * $p < 0.05$.

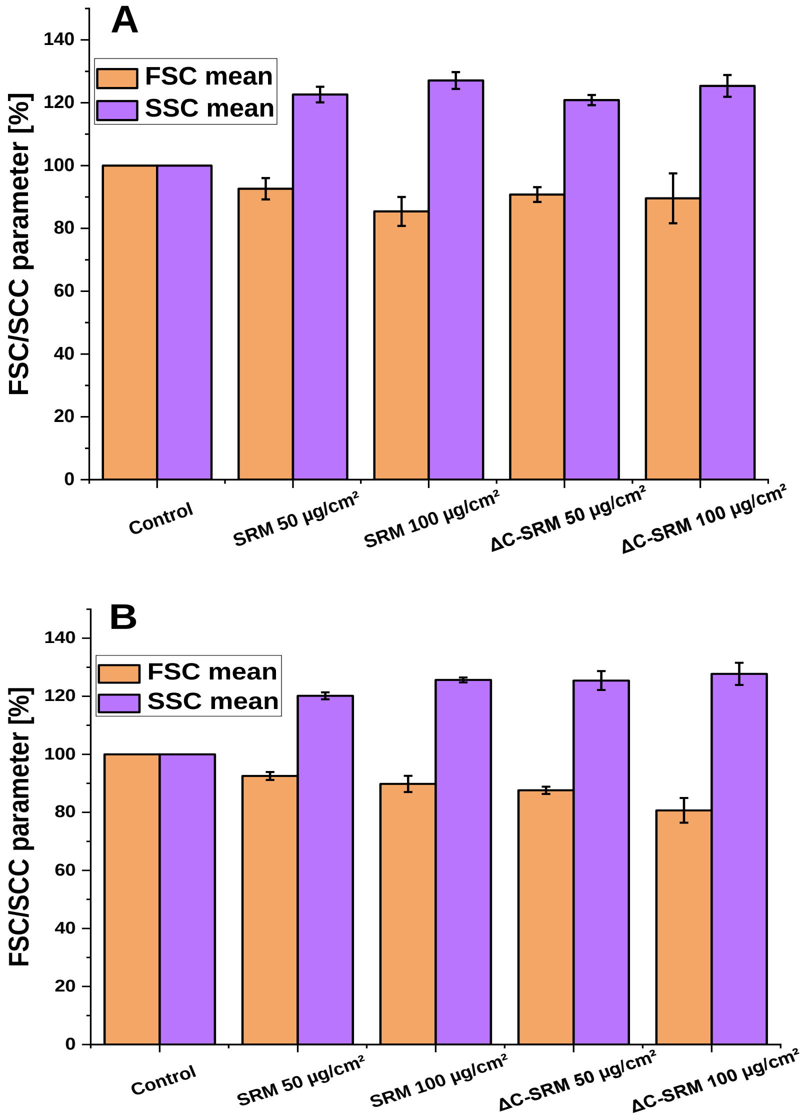

Fig. S4. The flow cytometry parameters (FSC and SSC) for A549 cells after 24 h exposure to different concentrations of PM samples implanted on an uncoated (A) or coated with collagen (B) surfaces. The results are representative of at least three independent experiments performed in triplicate and are expressed as mean ± SEM (error bars) of replicates.

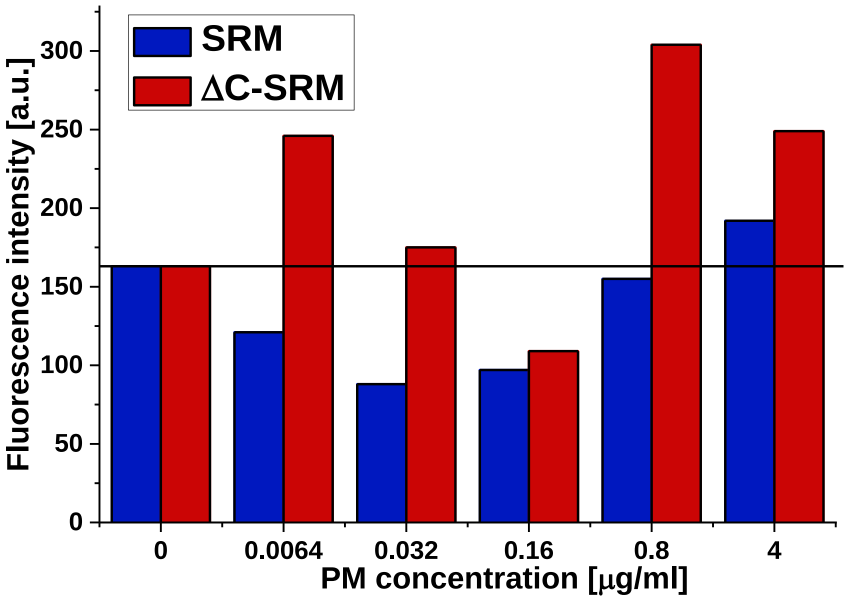

Fig. S5. The ROS species production in A549 cells after exposure to SRM/ΔC-SRM samples for 1 h evaluated using cyto-ID Hypoxia/Oxidative stress detection kit.

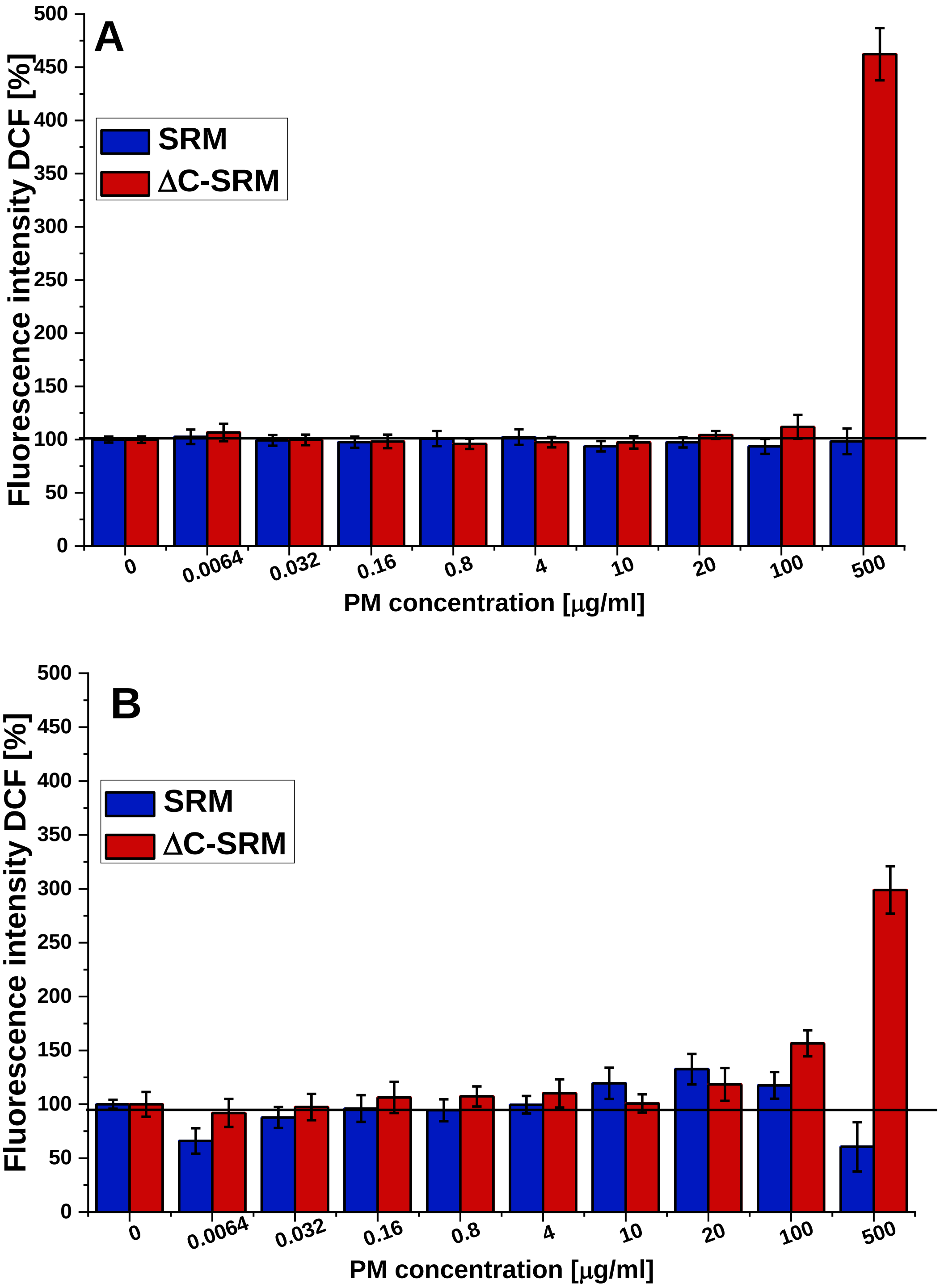

Fig. S6. The ROS production in A549 cells after exposure to SRM/ΔC-SRM samples for 24 (A) and 72 (B) hours measured using 2,7-dichlorodihydrofluorescein diacetate probe. The results are representative at least three independent experiments.

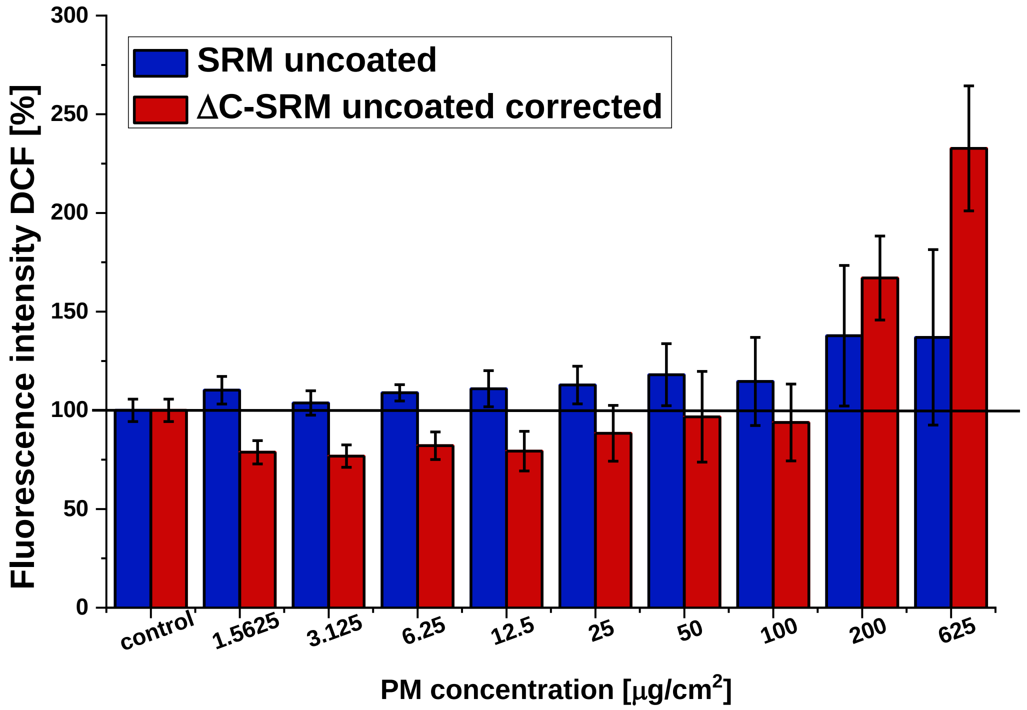

Fig. S7. The ROS production in A549 cells after 24 h exposure to different concentrations of PM samples implanted on an uncoated surface with ΔC-SRM sample mass decrease correction measured using 2,7-dichlorodihydrofluorescein diacetate assay.

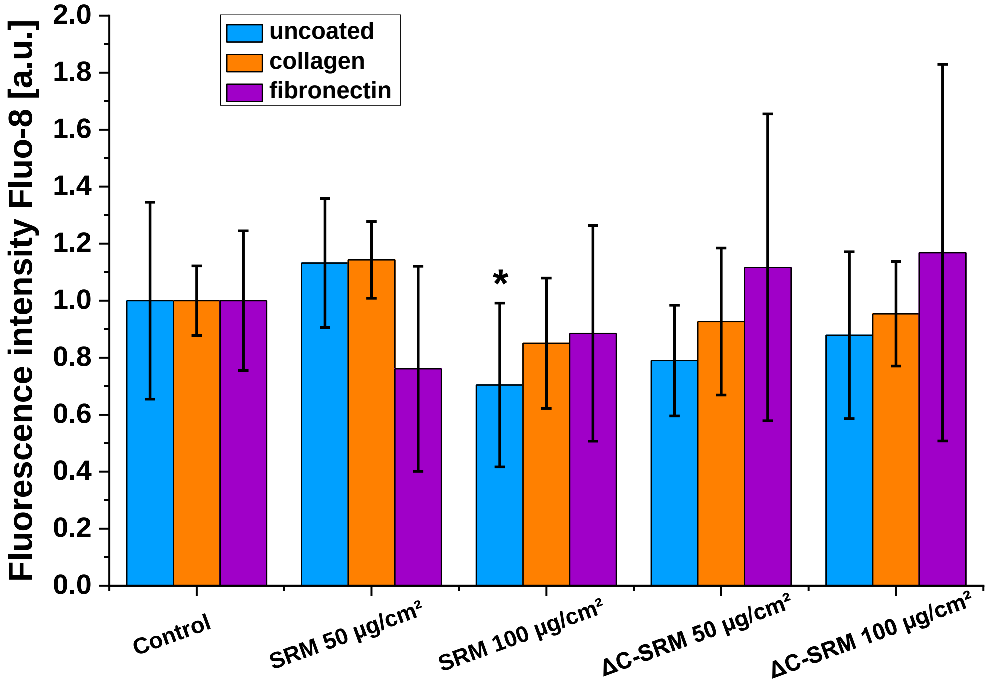

Fig. S8. The changes in cytosolic calcium level in A549 cells after 24 h incubation with different concentrations of PM-covered plates either uncoated or coated with fibronectin/collagen measured using

Fluo-8 AM probe. The results are representative of at least three independent experiments performed in triplicate and are expressed as mean ± SEM (error bars) of replicates, * $p < 0.05$.
